# Editorial: In the March 2012 issue

**DOI:** 10.1590/S1980-57642012DN06010001

**Published:** 2012

**Authors:** Ricardo Nitrini

**Affiliations:** Editor-in-Chief

In this issue we are publishing one review, seven original papers, two case reports, and brief reviews.

**Romann et al.** conducted a systematic review of the literature to identify the neuropsychological tests most commonly used for cognitive evaluation in individuals with Parkinson Disease undergoing deep brain stimulation. The Verbal Fluency test was found to be the most used instrument, followed by the Boston Naming Test.

**Go et al.** present their data showing that a simple visual magnetic resonance imaging rating scale can contribute relevant information on the neural correlates of behavioral disturbances in frontotemporal dementia and in Alzheimer's disease. This scale may be useful for the differential diagnosis of behavioral disturbances.

**Mello et al.** investigated young students with learning disabilities in single-day assessments including clinical-genetic exam, behavioral assessment and neuropsychological screening. This assessment method was able to define the diagnosis of several conditions and may be useful in clinical practice.

**Oliveira et al.** compared the performance of two age groups of adult individuals and observed that the younger adult group (19-39 years old) performed better than the middle-aged group (40-59 years old) in tasks predominantly involving processing speed, cognitive flexibility and lexical search.

**Lima-Silva et al.** investigated through a pilot case-control study whether training of old adults on activities related to executive functions had an impact on performance in tests of executive functions and on self-perception of functional performance.

**Jacinto et al.** evaluated the accuracy of simple tests in the screening of cognitive impairment by general internists. The combined use of the Category Fluency Test and Functional Activity Questionnaire showed high sensitivity and moderate specificity for screening cognitive impairment in the elderly.

**Mendes et al.** report four cases of severe cognitive impairment in early multiple sclerosis. After a review of the literature the authors concluded that this is a rare condition causing significant disability and may ultimately constitute another form of the disease.

**Martins et al.** compared patients with chronic post-traumatic headache against patients with migraine and controls for quality of life and presence of depressive and anxiety symptoms. Patients with chronic post-traumatic headache had lower quality of life and higher levels of anxiety and depressive symptoms compared to controls, but no difference was observed when patients with chronic post-traumatic headache were compared to patients with migraine.

**Listik et al.** report a case of acquired hepatocerebral degeneration emphasizing that this is an underdiagnosed condition characterized by parkinsonism, ataxia or other movement disorders and by neuropsychiatric and cognitive symptoms.

**Satler et al.** report a case of frontotemporal dementia associated with neurocysticercosis and comment on the relevance of this association to the clinical picture and evolution.

**Ricardo Nitrini** Editor-in-Chief

